# Exome Sequencing Identified Susceptible Genes for High Residual Risks in Early‐Onset Coronary Atherosclerotic Disease

**DOI:** 10.1002/clc.70066

**Published:** 2024-12-14

**Authors:** Runda Wu, Ya Su, Jianquan Liao, Juan Shen, Yuanji Ma, Wei Gao, Zheng Dong, Yuxiang Dai, Kang Yao, Junbo Ge

**Affiliations:** ^1^ Department of Cardiology, Zhongshan Hospital Fudan University Shanghai P.R. China; ^2^ Shanghai Institute of Cardiovascular Disease Shanghai P.R. China; ^3^ Department of Cardiology Zhongshan Hospital, Qingpu Branch Shanghai P.R. China; ^4^ Institute of Metagenomics, Qingdao‐Europe Advanced Institute for Life Sciences, BGI Research Qingdao P.R. China; ^5^ Department of Cardiology Nanjing Drum Tower Hospital Nanjing P.R. China; ^6^ NHC Key Laboratory of Viral Heart Diseases (Fudan University) Shanghai P.R. China; ^7^ Key Laboratory of Viral Heart Diseases Chinese Academy of Medical Sciences Beijing P.R. China; ^8^ National Clinical Research Center for Interventional Medicine Shanghai P.R. China

**Keywords:** early‐onset coronary atherosclerotic disease, exome sequencing, genetic risk, residual risk

## Abstract

**Aims:**

Despite the tremendous improvement in therapeutic medication and intervention for coronary atherosclerotic disease (CAD), residual risks remain. Exome sequencing enables identification of rare variants and susceptibility genes for residual risks of early‐onset coronary atherosclerotic disease (EOCAD) with well‐controlled conventional risk factors.

**Methods:**

We performed whole‐exome sequencing of subjects who had no conventional risk factors, defined as higher body mass index, smoking, hypertension and dyslipidemia, screened from 1950 patients with EOCAD (age ≤ 45 years, at least 50% stenosis of coronary artery by angiography), and selected control subjects from 1006 elder (age ≥ 65 years) with < 30% coronary stenosis. Gene‐based association analysis and clinical phenotypic comparison were conducted.

**Results:**

Subjects without defined conventional risk factors accounted for 4.72% of young patients. Totally, 6 genes might be associated with residual risk of EOCAD, namely *CABP1* (OR = 22.19, *p* = 0.02), *HLA‐E* (OR = 22.19, *p* = 0.02), *TOE1* (OR = 33.6, *p* = 0.002), *HPSE2* (OR = 11.1, *p* = 0.04), *CHST14* (OR = 22.19, *p* = 0.02) as well as *KLHL8* (OR = 22.19, *p* = 0.02). Phenotypic analysis displayed the levels of low‐density lipoprotein cholesterol in carriers of mutations from *CABP1*, *HLA‐E*, *TOE1*, and *HPSE2* were significantly elevated compared to noncarriers. Notably, extracellular matrix‐associated *CHST14* and fibrinogen‐associated *KLHL8* both displayed possible correlation with increased neutrophil proportion and decreased monocyte percentage (both *p* < 0.05), exerting potential effects on the residual inflammatory risks of EOCAD.

**Conclusion:**

The study identified six genes related to dyslipidemia and inflammation pathways with potential association with residual risk of EOCAD, which will contribute to precision‐based prevention in these patients.

**Trial Registration:**

The GRAND study was registered at www.clinicaltrials.gov on July 14, 2015, and the registry number is NCT 02496858.

## Graphical abstract

Whole exome sequencing were conducted in EOCAD patients without defined conventional risk factors and CAD‐free control subjects. The sequenced nonsynonymous single nucleotide variants were subjected to variant annotation and gene mapping with disease‐related database. Finally, six novel susceptible genes associated with residual risks for EOCAD were acquired by clinical phenotype analysis.

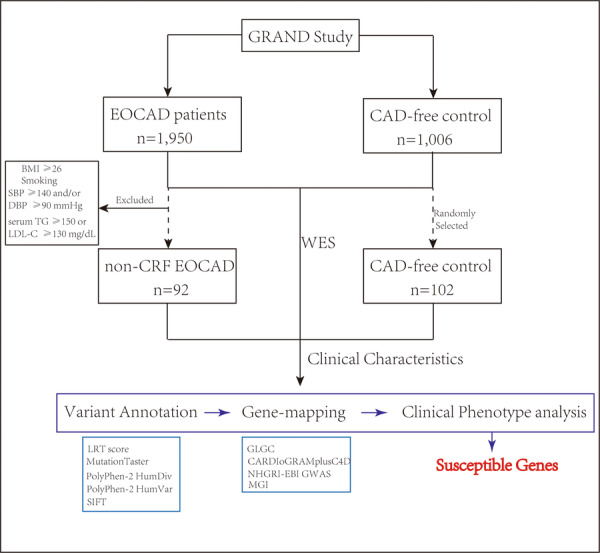

## 1 Introduction

Despite contemporary optimal medical and interventional therapies established for coronary atherosclerotic disease (CAD), patients retain at a high risk of adverse cardiovascular events whether after acute coronary syndrome or not [1, 2, 3]. Moreover, these unaddressed “residual risks” may especially exert impacts on patients with early‐onset coronary atherosclerotic diseases (EOCAD), which accounted for approximately 13% of the young population [4]. Patients with ST‐segment elevation myocardial infarction (STEMI) but fewer standard modifiable cardiovascular risk factors (SMuRFs) [5] accounted for 15%–17% of the overall patients with STEMI. These factors were also known as “conventional risk factors,” including hypertension (HTN), hypercholesterolemia, type 2 diabetes mellitus (T2DM) and smoking, while patients with fewer SMuRFs were reported to have an almost 50% higher 30‐day mortality rate than their counterparts with SMuRFs, indicating that there remains substantial disease burden in these patients. Specifically, while lipid‐lowering therapy has been extensively administered and low‐density lipoprotein cholesterol (LDL‐C) of patients with CAD have been controlled, the residual risk of adverse events remains high [2], which indicates that other unknown factors might be involved in atherosclerosis. Additionally, conventional risk factors could not accurately predict lesions burden in young patients [4], underlining a need to recognize residual risk factors for premature atherosclerosis to improve the precision‐based approaches to primary and secondary prevention [6].

Lipid components such as non‐high‐density lipoprotein cholesterol (non‐HDL‐C) [7, 8, 9, 10], which includes remnant‐like lipoprotein particles cholesterol (RLP‐C) [11, 12], along with particle size and concentration of high‐density lipoprotein (HDL) [13], apolipoprotein B [9, 10], lipoprotein (a) [14], triglyceride (TG) [15], and the logarithmic ratio of TG and HDL [16] have been implicated to increase the residual risk of cardiovascular events independently of LDL‐C levels. Besides, systemic and vascular inflammation has been considered to play a pivotal role in the pathogenesis of coronary atherosclerosis and progression of plaques [1]. It is reported that the lowering of high‐sensitivity C‐creative protein (hsCRP) by canakinumab for anti‐interleukin‐1 beta is crucial for reduction of cardiovascular events [17]. In addition to hsCRP [18], other inflammatory biomarkers may be associated with residual risks as well, for instance, urinary leukotrienes [19], perivascular fat attenuation index measured by coronary imaging [20], inflammasome complex [1] and so forth. Evidence is required regarding which critical factors have causal relationship with residual risk that accelerates atherosclerosis, which may be facilitated by human genetic studies.

We therefore performed whole exome sequencing (WES) to identify genes associated with residual risk of EOCAD in Chinese population, which will contribute to our understanding of pathogenesis of atherosclerosis and fuel the pipeline of cardiovascular drug discovery.

## 2 Methods

### 2.1 Study Population

We selected samples from cohort of the GRAND study, which is a prospective, multicenter, case–control study aimed to identify genetic factors related to EOCAD susceptibility and outcomes in Chinese [21]. Of the 1950 adult patients from the GRAND study with EOCAD (age ≤ 45 years) confirmed with evidence of at least 50% stenosis in one or more main coronary arteries by angiography, patients who had none of these conventional risk factors for CAD (referred to as “non‐CRF EOCAD” group), defined as body mass index (BMI) ≥ 26, previous and current smoking, diagnosed HTN (systolic blood pressure ≥ 140 mmHg and/or diastolic blood pressure ≥ 90 mmHg), serum TG ≥ 150 mg/dL or serum LDL‐C ≥ 130 mg/dL were enrolled between May 2017 and May 2018. In addition, randomly selected subjects with similar sample size from 1006 population‐based elder (age ≥ 65 years) control subjects with no angiographically significant CAD (< 30% coronary stenosis) from the GRAND study (named as “CAD‐free control” group) were also included. Coronary stenosis related to arteritis, myocardial bridge and transient coronary spasm were excluded. Baseline characteristics were extracted from multicenter databases of medical record. The study was approved by the central ethics committee of Zhongshan Hospital, Fudan University, and by institutional review boards and ethics committees at each participating medical center. All patients provided written informed consent and an independent data monitoring board reviewed the data at regular intervals.

### 2.2 Whole Exome Sequencing

Genomic DNA was isolated form whole blood samples and DNA libraries were prepared according to the manufacturers’ instructions. DNA samples were sequenced using a BGISEQ‐500 platform at Beijing Genomics Institute (BGI) with a Sure Select XT Human All Exon v5 array (Agilent, CA, USA). After raw reads were filtered by removing adapters and low‐quality sequences, the remaining clean reads were aligned to reference genome build GRCh37 with the Burrows‐Wheeler Aligner (BWA) [22] software (0.75). Duplicated reads were marked by Picard (https://broadinstitute.github.io/picard/, 2.10.10). Local realignment around indels and base quality score recalibration was performed on BAM files using the Genome Analysis Toolkit (GATK 3.2.2) [23]. To detect single‐nucleotide variants (SNVs) and small indels, GATK HaplotypeCaller was used following the best practices recommended. A series of quality control steps were applied and variants with a minor allele average depth < 4, an average depth < 8, a low mapping quality score, strand bias or allelic imbalance, or deviations from Hardy‐Weinberg equilibrium were excluded.

### 2.3 Variant Annotation

To identify the most possible rare and deleterious SNVs (minor allele frequency [MAF] < 5%), five protein prediction algorithms from ANNOVAR [24] (2017) (LRT score, MutationTaster, PolyPhen‐2 HumDiv, PolyPhen‐2 HumVar, and SIFT) were applied in non‐synonymous SNVs. We defined five SNV groups, including (1) nonsynonymous set (missense, splicing, nonsense, and indel frameshift variants); (2) a “deleterious (PolyPhen)” set consisting of missense annotated as “possibly damaging” or “probably damaging” by PolyPhen‐2 HumDiv software, nonsense, splice‐site, and indel frameshift; (3) a “deleterious (broad)” set consisting of missense annotated as “deleterious” by at least one of the five protein prediction algorithms, nonsense, splice‐site, and indel frameshift; (4) a “deleterious (strict)” set consisting of missense annotated as “deleterious” by all five protein prediction algorithms, nonsense, splice‐site, and indel frameshift; and (5) “disruptive” mutations only (nonsense, splice‐site or indel frameshift). Of all qualified variants, we used Collapse (two‐tailed Fisher's exact test by R 3.6.1 software) to perform gene‐based association analysis on these five defined SNV sets (MAF < 5%) in the combined population (non‐CRF EOCAD cases and CAD‐free control). The threshold of *p* value at 0.05 and odds ratio (OR) at 3.5 was applied, and 844 genes (*p* < 0.05 and OR > 3.5) from the five SNV sets were screened as initial candidate genes associated with EOCAD incidence but without conventional risk factors in the further analysis.

### 2.4 Gene‐Mapping With Disease‐Related Database

Phenotypic annotation of these genes were conducted with evidence of influencing circulating lipids from database of Global Lipids Genetics Consortium (GLGC) Results (http://csg.sph.umich.edu/willer/public/lipids2013/), evidence of correlation with incidence of CAD from database of the CARDIoGRAMplusC4D Consortium (http://www.cardiogramplusc4d.org/), evidence of five related phenotypes (cardiovascular+ adipose+ metabolism+ endocrine/exocrine +liver) verified in mouse genome informatics (MGI, http://www.informatics.jax.org), and evidence of association with incidence of CAD from the NHGRI‐EBI GWAS catalog (https://www.ebi.ac.uk/gwas/docs/file-downloads), and genes were further mapped and filtered with phenotypes above.

### 2.5 Clinical Phenotype Analysis

In gene level, linear regression (by R 3.6.1) was performed to delineate the difference of clinical features between carriers of rare mutations and noncarriers in non‐CRF EOCAD cases.

### 2.6 Data Availability

The WES data in the GRAND study have been deposited in the China National GeneBank databases under the accession code CNP0000730 following the regulations of the Human Genetic Resources Administration of China (HGRAC). Data of this study are available from the corresponding authors upon reasonable request and approval of the HGRAC.

## 3 Results

### 3.1 Clinical Characteristics of Subjects

We screened from 1950 patients with angiographical diagnosis of EOCAD enrolled from May 2017 to May 2018, and 92 patients with none of defined conventional risk factors were included, which accounted for 4.72% of young patients. Clinical characteristics are summarized in Table 1. The mean age of patients in the non‐CRF EOCAD group was 39 years while CAD‐free control subjects were averagely 71 years of age. In non‐CRF EOCAD group, patients were mostly male (72.82%), with a significantly lower BMI (22.88 ± 1.90 vs. 24.14 ± 3.36 in CAD‐free control, *p* < 0.001), lower systolic blood pressure (SBP) (122 ± 14.78 mmHg vs. 133.89 ± 19.84 mmHg, *p* < 0.0001), lower levels of TG (86.62 ± 28.27 mg/dL vs. 137.97 ± 100.17 mg/dL, *p* < 0.0001), lower levels of LDL‐C (76.03 ± 33.03 mg/dL vs. 96.04 ± 46.56 mg/dL, *p* < 0.0001), and no record of previous and current smoking. In addition to controlled conventional risk factors of overweight, HTN, hypertriglyceridemia, high LDL‐C and smoking, proportion of patients with T2DM were significantly lower in the non‐CRF EOCAD group (5.43%) than CAD‐free control (17.99%, *p* = 0.022), so was circulating level of hemoglobin A1c (HbA1c, 5.572 ± 0.81% vs. 6.720 ± 5.38%, *p* < 0.0001). Other compositions of serum lipids, including ApoA1, ApoB, ApoE and NHDL‐C, showed markedly lower levels (all *p* < 0.001) in patients with EOCAD, which is in line with the low levels of TG and LDL‐C.

**Table 1:** Clinical characteristics of subjects with non‐CRF EOCAD and CAD‐free control.

Characteristics	non‐CRF EOCAD (*n* = 92)	CAD‐free Control (*n* = 102)	*p* value
Male (%)	72.8	44.4	< 0.0001
Age (years)	39.0 (4.5)	71.0 (5.3)	< 0.0001
BMI (kg/m^2^)	22.9 (1.9)	24.1 (3.4)	< 0.001
Body surface area (m^2^)	1.7 (0.16)	NA	NA
SBP (mmHg)	122.0 (14.78)	133.9 (19.84)	< 0.0001
DBP (mmHg)	77.3 (11.87)	76.0 (11.28)	0.268
Heart rate (bpm)	75.1 (11.62)	76.3 (12.81)	0.213
LVEF (%)	60 (9.43)	63 (8.09)	< 0.05
LVDD (mm)	47.6 (5.96)	48.1 (7.01)	0.652
LVSD (mm)	30.1 (9.25)	31.6 (6.77)	0.797
Hemoglobin (g/L)	138.9 (15.72)	130.4 (15.64)	< 0.0001
RBC count (10^12^/L)	4.7 (0.50)	4.3 (1.15)	< 0.0001
WBC count (10^9^/L)	7.1 (2.62)	6.0 (1.74)	< 0.0001
Platelet count (10^9^/L)	234.4 (62.92)	191.2 (58.04)	< 0.0001
Neutrophil in WBC (%)	63.4 (11.60)	60.0 (9.84)	< 0.05
Lymphocyte in WBC (%)	27.3 (10.02)	29.3 (8.87)	0.093
Monocyte in WBC (%)	7.0 (2.45)	7.8 (3.97)	< 0.05
RBC distribution width (%)	12.8 (1.17)	13.2 (2.71)	< 0.001
Platelet distribution width (%)	13.9 (2.89)	13.7 (2.67)	0.716
ALT (U/L)	28.3 (18.80)	24.2 (30.50)	< 0.01
AST (U/L)	33.5 (48.70)	24.7 (26.97)	0.310
Creatine (umol/L)	72.7 (15.82)	79.0 (53.75)	0.366
Uric acid (umol/L)	335.9 (91.59)	351.5 (100.35)	0.275
Fasting glucose (mmol/L)	5.6 (1.98)	5.9 (2.25)	0.125
HbA1c (%)	5.6 (0.81)	6.7 (5.38)	< 0.0001
TC (mg/dL)	132.5 (36.29)	157.1 (37.20)	< 0.0001
TG (mg/dL)	86.6 (28.27)	138.0 (100.17)	< 0.0001
HDL‐C (mg/dL)	42.0 (8.36)	46.3 (13.23)	< 0.05
LDL‐C (mg/dL)	76.0 (33.03)	87.6 (40.10)	< 0.0001
ApoA1 (g/L)	1.2 (0.19)	1.3 (0.27)	< 0.001
ApoB (g/L)	0.6 (0.20)	0.8 (0.23)	< 0.0001
ApoE (mg/L)	28.3 (9.99)	40.0 (14.07)	< 0.0001
NHDL‐C (mmol/L)	2.4 (0.94)	2.9 (0.92)	< 0.0001
CK (U/L)	144.8 (210.04)	90.8 (195.42)	< 0.001
cTnI (ng/mL)	4.4 (12.18)	0.1 (0.38)	< 0.001
Fibrinogen (g/L)	3.8 (4.61)	2.8 (0.79)	< 0.05
CRP (mg/L)	5.5 (16.22)	4.6 (14.97)	< 0.001
PT (s)	16.9 (39.65)	12.3 (4.65)	< 0.0001
INR	1.0 (0.10)	1.1 (0.43)	< 0.01
APTT (s)	31.6 (14.03)	27.3 (4.08)	< 0.0001
D‐Dimer (mg/L)	0.2 (0.18)	0.5 (0.90)	< 0.0001
Acute coronary syndrome (%)	41.3	0	NA
Unstable angina (%)	23.9	0	NA
ST‐segment elevation MI (%)	13.0	0	NA
Non‐ST‐segment elevation MI(%)	4.3	0	NA
Previous history of HTN (%)	0	57.8	NA
Previous history of T2DM (%)	5.4	18.0	< 0.05
Previous history of MI (%)	23.1	2.5	< 0.0001
Multivessel coronary lesions (%)	8.7	NA	NA
Lipid‐lowering drugs (%)	17.4	21.8	0.424
Antidiabetic drugs (%)	3.1	14.7	0.107
Previous and current smoking (%)	0	NA	NA
Family history of CAD (%)	2.5	NA	NA

*Note:* Quantitative data and categorical data are presented as mean ± SD and percentage with Wilcoxon test and Fisher's exact test, respectively.

Abbreviations: ALT, alanine transaminase; ApoA1, apolipoprotein A1; ApoB, apolipoprotein B; ApoE, apolipoprotein E; APTT, activated partial thromboplastin time; AST, aspartate transaminase; BMI, body mass index; CAD, coronary artery disease; CK, creatine kinase; CRP, C reactive protein; cTnI, cardiac troponin I; DBP, diastolic blood pressure; EOCAD, early‐onset coronary artery disease; HbA1c hemoglobin A1c; HDL‐C, high‐density lipoprotein‐cholesterol; HTN, hypertension; INR, international normalization ratio; LDL‐C, low‐density lipoprotein‐cholesterol; LVDD, left‐ventricular diastolic diameter; LVEF, left‐ventricular ejection fraction; LVSD, left‐ventricular systolic diameter; MI, myocardial infarction; NA, not applicable; NHDL‐C, non‐HDL‐cholesterol; Non‐CRF, with none of the defined conventional risk factors; PT, prothrombin time; RBC, red blood cell; SBP, systolic blood pressure; T2DM, type 2 diabetes mellitus; TC, total cholesterol; TG, triglyceride; WBC, white blood cell.

At the time of diagnosis of CAD, more patients (23.08%) had previous myocardial infarction (MI) in the non‐CRF EOCAD group, compared to only 2.51% in CAD‐free control (*p* < 0.0001). Patients with EOCAD displayed significantly higher levels of biomarkers of cardiac injury on admission such as cardiac troponin I (cTnI, *p* < 0.001), creatine kinase (CK, *p* < 0.001) and alanine transaminase (ALT, *p* < 0.01), which was in concordance with the high incidence of ACS in these young patients, despite the unspecific diagnostic feature of CK and ALT during cardiac insult. As acute‐phase reactant protein, fibrinogen is associated with injury and stress responses [25], and patients with EOCAD showed significantly higher fibrinogen than control (3.805 ± 4.61 g/L vs. 2.779 ± 0.79 g/L, *p* < 0.05), along with higher CRP levels (5.515 ± 16.22 mg/L vs. 4.593 ± 14.97 mg/L, *p* < 0.001). Moreover, it was demonstrated that inflammatory response‐related total white blood cell (WBC) count (*p* < 0.0001) and proportion of neutrophils (*p* < 0.05) were slightly elevated in the EOCAD group, with the percentage of monocytes mildly decreased (*p* < 0.05).

### 3.2 Gene‐Based Association Analysis for Residual Risk of Eocad

WES was performed in cases with non‐CRF EOCAD and CAD‐free control, and an average coverage of 133.5‐fold on target and 98% of bases covered at ≥ 20× were achieved. A total of 1 116 161 variants were got during the WES analysis across all samples in exonic and its extended regions (200 bp), including 66 272 indels and 1 049 889 SNVs. To identify causative genes associated with residual risk of EOCAD, gene‐based association tests were implemented with rare coding variants on the five defined SNV sets (MAF of < 5%, results shown in Table S1). Of the 844 genes (*p* < 0.05 and OR > 3.5) screened as initial candidate genes from the five SNV sets (Table S2), 336 genes were associated with residual risk of CAD, evidence found in at least one databases of GLGC, CARDIoGRAMplusC4D and MGI (Table S3). Furthermore, those genes were excluded which did not reach significance during the phenotypic comparison between carriers and non‐carriers of mutations in the genes within non‐CRF EOCAD group, leaving 273 genes valid (*p* < 0.05) for specific phenotype analysis afterwards (Table S4). Notably, two genes with specific phenotypes were mentioned in three databases above and in GWAS catalog, namely *QTRT1* discovered in two SNV sets and *LDLR* discovered in three SNV sets (Tables 2 and S5). In detail, carriers with mutations of *LDLR* had higher uric acid (linear regression beta = 85.12, *p* = 0.01), LDL‐C (beta = 31.4, *p* = 0.013) and ApoB (beta = 0.18, *p* = 0.035) when compared to noncarriers, while carriers of mutations in *QTRT1* showed higher uric acid (beta = 83.15, *p* = 0.017), TG (beta = 32.19, *p* = 0.005) and d‐dimer (beta = 0.23, *p* = 0.002).

**Table 2:** Phenotypic association analysis of two genes all mentioned in GLGC, CARDIoGRAMplusC4D, MGI, and GWAS catalog.

Mutation set	Gene	cMAF	OR	*p* value	PDW P (Beta)	UA P (Beta)	TG P (Beta)	LDL‐C P (Beta)	ApoB P (Beta)	D‐dimer P (Beta)
Nonsynoymous	*LDLR*	0.027	4.25	0.002	0.062 (2.16)	0.01 (85.12)^a^	0.62 (−5.29)	0.013 (31.4)^a^	0.035 (0.18)^a^	0.156 (−0.1)
Broad	*LDLR*	0.076	4.27	0.004	0.009 (3.21)^a^	0.025 (80.24)^a^	0.21 (−14.25)	0.109 (22.04)	0.069 (0.18)	0.124 (−0.11)
PolyPhen	*LDLR*	0.019	4.60	0.006	0.001 (4.24)^a^	0.167 (54.06)	0.346 (−11.45)	0.356 (13.59)	0.081 (0.18)	0.121 (−0.12)
Broad	*QTRT1*	0.017	4.05	0.009	0.662 (−0.53)	0.017 (83.15)^a^	0.005 (32.19)^a^	0.693 (5.7)	0.5 (0.06)	0.002 (0.23)^a^
PolyPhen	*QTRT1*	0.014	4.06	0.017	0.974 (−0.04)	0.112 (61.15)	0.02 (29.48)^a^	0.742 (5.17)	0.773 (0.03)	0.406 (0.07)

*Note:* OR and *p* value were calculated by Fisher's exact test. Beta (P) represents the coefficient and *p* value of effect of the mutation set in genes on the corresponding clinical phenotypic markers in linear regression.

Abbreviations: ApoB, apolipoprotein B; cMAF, collapsed minor allele frequency; LDL‐C, low‐density lipoprotein‐cholesterol; OR, odds ratio; PDW, platelet distribution width; TG, triglyceride; UA, uric acid.

^a^ Refers to significant results.

### 3.3 Gene‐Based Association Analysis for Risk of Hyperlipidemia

Among the 273 candidate genes, 42 genes were mentioned in the GLGC database (Table S6), which demonstrated potential correlation with significant alterations of serum TC, TG, LDL‐C and HDL‐C. To be specific, 7 genes from different SNV sets which were mapped with evidence of influencing the level of LDL‐C from the GLGC (Table 4), were implicated in significant change of LDL‐C (Table S7), and only three genes, *PPARD*, *CABP1*, and *LDLR* showed positive association with serum LDL‐C (Table 3). Given the elaborate and enormous studies on the function and regulation of *LDLR* gene [26], the effect size of *PPARD* (OR = 22.19, *p* = 0.02) and *CABP1* (OR = 22.19 *p* = 0.02) on risk of EOCAD and on serum LDL‐C (beta = 48.64 and 73.87, respectively, *p* < 0.05) was prominent and of interest. Besides, *PPARD* also displayed significant association with serum ApoA1 and ApoB, despite lower effect size (beta = 0.33 for ApoA1, beta = 0.31 for ApoB, *p* < 0.05). Another gene, *HLA‐E* was found to be positively correlated with serum TC (beta = 87.86, *p* < 0.05) and LDL‐C (beta = 96.49, *p* < 0.05), which was mentioned in the GLGC as well (Table S8). *ZNF101* was found to be positively correlated with serum TG (beta = −31.19, *p* < 0.05), which was mentioned in the GLGC as well (Table S9). In addition, mild impact (beta < 5) on the circulating level of other lipid composition assumed as residual risk factors including ApoA1, ApoB, ApoE and NHDL‐C was shown in several genes without any evidence mapped in the GLGC (Tables 3, 4, and S10). These genes included *PPARD, LDLR, GBA, ADGRA2, SPRYD3, RASGEF1B, TOE1, APBB2, LTF, TP53BP1, FGB, HPSE2, MNS1*, and *PM20D1*. However, *LTF* (beta = 16.23, *p* < 0.05) and *TP53BP1* (beta = 18.47, *p* < 0.05) exerted relatively strong correlation with serum ApoE, and *TOE1* was in turn found to potentially increase the TC and LDL‐C (beta = 68.66 and 62.05, both *p* < 0.05) in carriers with mutations, so was gene *HPSE2* (beta = 52.78 and 41.63, both *p* < 0.05).

**Table 3:** Phenotypic association analysis of genes related to residual hyperlipidemia which were also mentioned in the GLGC.

Mutation set	Gene	cMAF	OR	*p* value	TC P (Beta)	LDL‐C P (Beta)	ApoA1 P (Beta)	ApoB P (Beta)	ApoE P (Beta)	NHDL‐C P (Beta)
Nonsynoymous	*PPARD*	0.003	22.19	0.02	0.075 (47.6)	0.045 (48.64)^a^	0.019 (0.33)^a^	0.03 (0.31)^a^	NA	0.148 (1.02)
Broad	*PPARD*	0.003	22.19	0.02	0.075 (47.6)	0.045 (48.64)^a^	0.019 (0.33)^a^	0.03 (0.31)^a^	NA	0.148 (1.02)
PolyPhen	*PPARD*	0.003	22.19	0.02	0.075 (47.6)	0.045 (48.64)^a^	0.019 (0.33)^a^	0.03 (0.31)^a^	NA	0.148 (1.02)
Nonsynoymous	*CABP1*	0.003	22.19	0.02	0.121 (58.38)	0.03 (73.87)^a^	0.843 (−0.04)	0.133 (0.31)	NA	NA
Broad	*CABP1*	0.003	22.19	0.02	0.121 (58.38)	0.03 (73.87)^a^	0.843 (−0.04)	0.133 (0.31)	NA	NA
Nonsynoymous	*LDLR*	0.027	4.25	0.002	0.363 (12.94)	0.013 (31.4)^a^	0.518 (−0.06)	0.035 (0.18)^a^	0.691 (−2.5)	0.343 (0.44)
Nonsynoymous	*HLA‐E*	0.003	22.19	0.02	0.017 (87.86)^a^	0.004 (96.49)^a^	NA	NA	NA	0.224 (1.18)
Broad	*HLA‐E*	0.003	22.19	0.02	0.017 (87.86)^a^	0.004 (96.49)^a^	NA	NA	NA	0.224 (1.18)
Strict	*GBA*	0.003	22.19	0.02	0.494 (18.42)	0.977 (−0.7)	0.005 (0.39)^a^	0.889 (0.02)	NA	0.797 (−0.18)
Strict	*ADGRA2*	0.004	11.1	0.04	0.847 (5.19)	0.534 (−15.14)	0.016 (0.33)^a^	0.223 (−0.18)	0.65 (−4.79)	0.245 (−0.81)
Nonsynoymous	*SPRYD3*	0.004	11.1	0.04	0.355 (24.8)	0.327 (23.91)	0.524 (−0.13)	0.027 (0.44)^a^	NA	0.154 (1.38)
PolyPhen	*RASGEF1B*	0.004	11.1	0.04	0.183 (49.72)	0.124 (52.11)	0.524 (−0.13)	0.027 (0.44)^a^	NA	0.154 (1.38)
Strict	*TOE1*	0.004	33.6	0.002	0.009 (68.66)^a^	0.01 (62.05)^a^	0.102 (0.23)	0.017 (0.34)^a^	0.779 (−2.14)	0.002 (2.13)^a^
Strict	*APBB2*	0.004	11.1	0.04	0.183 (49.72)	0.124 (52.11)	0.524 (−0.13)	0.027 (0.44)^a^	NA	0.154 (1.38)
Strict	*LTF*	0.012	5.02	0.02	0.931 (1.93)	0.9 (−2.54)	0.97 (−0.004)	0.02 (−0.27)^a^	0.028 (16.23)^a^	0.447 (−0.44)
Strict	*TP53BP1*	0.006	8.41	0.02	0.24 (−25.64)	0.1 (−32.51)	0.693 (0.05)	0.491 (−0.08)	0.011 (18.47)^a^	0.175 (−0.95)
Nonsynoymous	*FGB*	0.019	4.6	0.006	0.984 (−0.39)	0.726 (−6.17)	0.246 (−0.12)	0.758 (0.03)	0.782 (1.53)	0.029 (0.99)^a^
Broad	*FGB*	0.019	4.6	0.006	0.984 (−0.39)	0.726 (−6.17)	0.246 (−0.12)	0.758 (0.03)	0.782 (1.53)	0.029 (0.99)^a^
Nonsynoymous	*HPSE2*	0.009	4.8	0.04	0.016 (52.78)^a^	0.037 (41.63)^a^	0.451 (0.11)	0.129 (0.23)	0.884 (−1.6)	0.034 (1.45)^a^
Broad	*HPSE2*	0.008	5.6	0.03	0.016 (52.78)^a^	0.037 (41.63)^a^	0.451 (0.11)	0.129 (0.23)	0.884 (−1.6)	0.034 (1.45)^a^
PolyPhen	*HPSE2*	0.004	11.1	0.04	0.001 (81.96)^a^	0.005 (66.3)^a^	0.512 (0.13)	0.169 (0.28)	NA	0.034 (1.45)^a^
Disruptive	*MNS1*	0.003	22.19	0.02	0.825 (8.33)	0.846 (−6.63)	0.37 (0.18)	0.782 (−0.06)	0.791 (−2)	0.048 (1.36)^a^
Disruptive	*PM20D1*	0.004	11.1	0.04	0.119 (−60.27)	0.142 (−51.64)	0.882 (0.03)	0.162 (−0.29)	0.605 (−5.53)	0.003 (2.87)^a^
Broad	*SLK*	0.008	5.6	0.03	0.621 (19.49)	0.561 (20.84)	0.135 (0.31)	0.474 (−0.15)	0.605 (−5.53)	0.003 (2.87)^a^
PolyPhen	*SLK*	0.004	33.6	0.002	0.621 (19.49)	0.561 (20.84)	0.135 (0.31)	0.474 (−0.15)	0.605 (−5.53)	0.003 (2.87)^a^

*Note:* OR and *p* value were calculated by Fisher's exact test. Beta (P) represents the coefficient and *p* value of effect of the mutation set in genes on the corresponding clinical phenotypic markers in linear regression.

Abbreviations: ApoA1, apolipoprotein A1; ApoB, apolipoprotein B; ApoE, apolipoprotein E; cMAF, collapsed minor allele frequency; LDL‐C, low‐density lipoprotein‐cholesterol; NA, not applicable; NHDL‐C, non‐HDL‐cholesterol; OR, odds ratio; TC, total cholesterol.

^a^ Refers to significant results.

**Table 4:** Genes associated with residual hyperlipidemia and association with four types of lipids from the GLGC.

Gene	TC‐P value	TG‐P value	LDL‐C‐P value	HDL‐C‐P value
*QTRT1*	3.65 × 10^−16^	NA	2.18 × 10^−19^	NA
*PPARD*	1.61 × 10^−7^	NA	5.65 × 10^−6^	NA
*CABP1*	NA	NA	2.87 × 10^−5^	NA
*LDLR*	5.43 × 10^−202^	NA	3.85 × 10^−262^	6.32 × 10^−5^
*HLA‐E*	5.15 × 10^−7^	1.35 × 10^−9^	NA	4.01 × 10^−5^
*GBA*	NA	NA	NA	NA
*ADGRA2*	NA	NA	NA	NA
*SPRYD3*	NA	NA	NA	NA
*RASGEF1B*	NA	NA	NA	NA
*TOE1*	NA	NA	NA	NA
*APBB2*	NA	NA	NA	NA
*LTF*	NA	NA	NA	NA
*TP53BP1*	NA	NA	NA	NA
*FGB*	NA	NA	NA	NA
*HPSE2*	NA	NA	NA	NA
*MNS1*	NA	NA	NA	NA
*PM20D1*	NA	NA	NA	NA
*SLK*	NA	NA	NA	NA

*Note: p* value was calculated from data exported from the GLGC (http://csg.sph.umich.edu/willer/public/lipids2013/).

Abbreviation: NA, not applicable.

### 3.4 Gene‐Based Association Analysis for Risk of Bleeding and Thrombosis

In spite of the higher levels of PT and APTT which indicate relatively higher bleeding tendency in the non‐CRF EOCAD group (16.92 ± 39.65 vs. 12.28 ± 4.65 in PT level of CAD‐free control, 31.58 ± 14.03 vs. 27.29 ± 4.08 in APTT of control, both *p* < 0.0001), we attempted to detect any genes associated with potential thrombosis which corresponded to reduction of PT and APTT. Nineteen genes were acquired from the filtration of significance of difference among 273 candidate genes (Table S11), and finally 7 genes revealed both significant alterations of PT and APTT (Table S12). Whereas these genes (*FGB, C4orf45, SIK2, NPAS3*, and *SETD6*) showed markedly increment of both PT and APTT, therefore imposing no thrombotic risk but higher bleeding risk, which was less likely to contribute to the residual risk of EOCAD.

### 3.5 Gene‐Based Association Analysis for Risk of Inflammation

Likewise, we focused on the genes associated with residual inflammatory risk of EOCAD. As to the inflammatory CRP, 22 genes from SNV sets were identified to augment the level of CRP in carriers of mutations of these genes (Table S13), compared to 22 genes likely to raise the level of fibrinogen (Table S14), which displayed divergent distribution of genes and no intersection between these two sets of genes. Owing to the phenotypic difference abovementioned that WBC count (*p* < 0.0001) and proportion of neutrophils (*p* < 0.05) of young patients with EOCAD group were higher than control, accompanied with lower proportion of monocytes (*p* < 0.05), we filtered genes potentially boosting CRP with corresponding significantly increased counts of WBCs and neutrophils and fewer monocytes, and discovered two genes (*FBLN5* and *PCSK5*, Extended Table 1). In contrast, among 22 genes correlated with increment of fibrinogen, merely one gene, *KLHL8* (beta for fibrinogen 9.94, *p* = 0.003) was found to positively impact WBC count (beta = 4.91, *p* = 0.01) and neutrophil percentage (beta = 16.9, *p* < 0.05), with a reverse correlation with monocyte percentage (beta = −5.17, *p* = 0.002), coinciding with the phenotypic alterations in non‐CRF EOCAD group. Additionally, genes associated with elevation of neutrophils (beta > 0 and *p* < 0.05, Table S15) also showed association with higher WBC counts (*RALY*) and decline of proportions of lymphocytes and monocytes (*TOP2A* and *CHST14*), and only decreased monocyte proportion (*C2orf73*).

## 4 Discussion

In our study, we found 4.72% of patients with EOCAD having no defined conventional risk factors (overweight, HTN, hyperglyceridemia, high levels of LDL‐C, and smoking). Gene‐based association test combined with the clinical phenotype analysis and data extracted from four CAD‐related databases, a total of 273 candidate genes with significant difference between carriers of rare mutations and non‐carriers from the non‐CRF EOCAD group were identified, with matched evidence from at least one database. In line with previous studies, the major lesion type of patients with EOCAD was single‐vessel lesion, and the incidence of ACS, circulating cardiac troponin, acute phase reactant protein and inflammatory biomarkers were higher in the EOCAD group than control.

**Figure 1:**
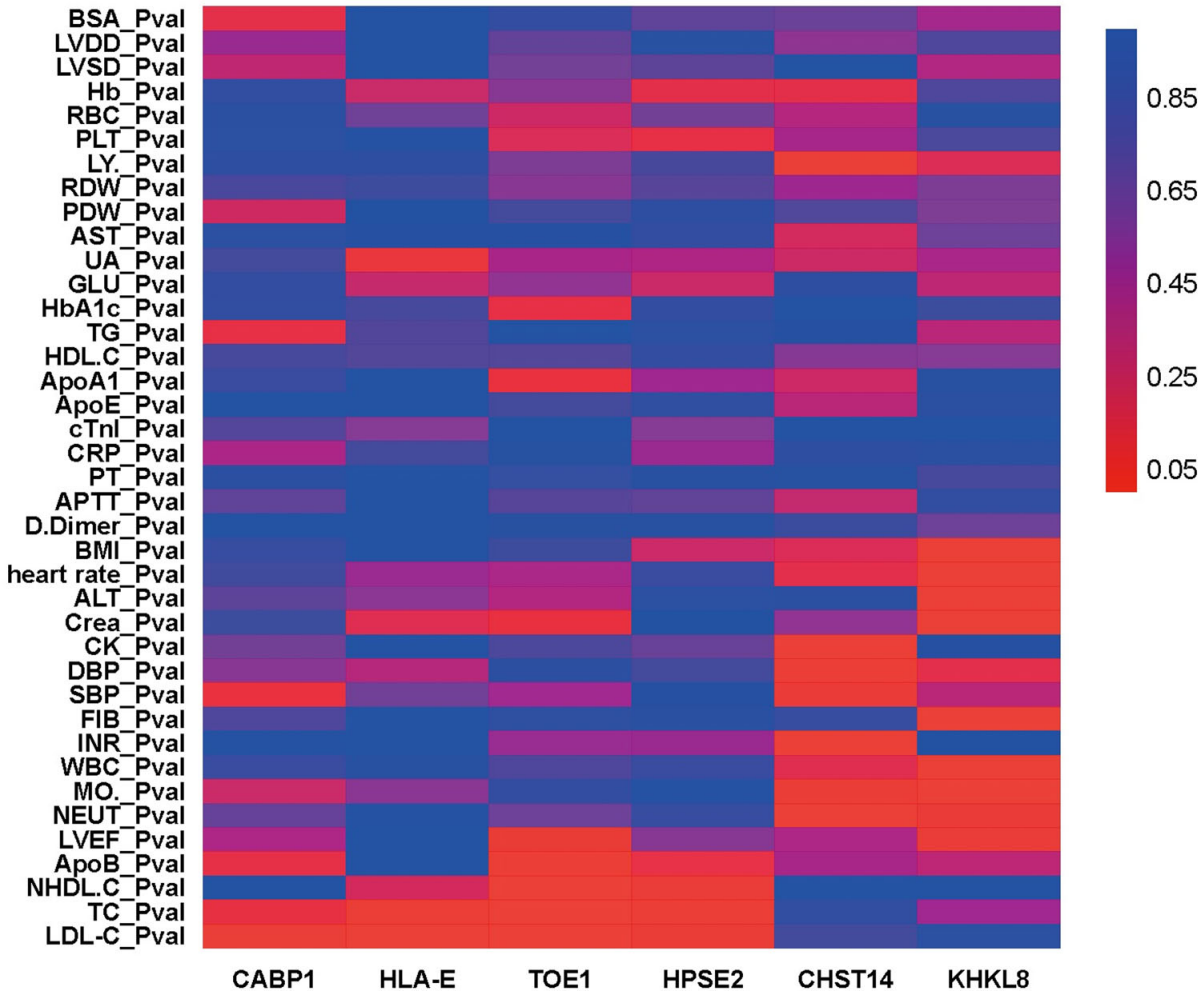
Heatmap of *CABP1, HLA‐E, TOE1, HPSE2, CHST14*, and *KHKL8* associated with clinical phenotypes. Data were shown as p values calculated by linear regression analysis. *p* < 0.05 was considered significant difference of certain phenotype between carriers of rare mutations in the candidate genes and noncarriers from the EOCAD group.

To assess residual genetic risk of patients in the non‐CRF EOCAD group, genes correlated with raised conventional risk factors were excluded. Although elevated low‐density lipoprotein cholesterol and hypertriglyceridemia were used as exclusion criteria in our study, other lipid factors were not excluded from conrtributing to the morbidity of non‐CRF‐EOCAD group. For example, Lp(a), small dense LDL (sdLDL) and other lipid factors have been found to be related to the onset of coronary atherosclerotic disease [27, 28]. Therefore, we further used the lipid database to conduct a correlation study with the aim of discovering new genes associated with residual lipid factors. Numerous genes related to lipid metabolism and dyslipidemia were determined, apart from the well‐studied *LDLR*, such as *QTRT1*, *PPARD*, *CABP1*, *HLA‐E, TOE1*, and *HPSE2* with correlation with higher TC or LDL‐C, versus *LTF* and *TP53BP1* with higher ApoE. Among these genes, *QTRT1*, *PPARD*, *CABP1*, *HLA‐E* were implicated in alterations of serum TC, TG, LDL‐C and HDL‐C. Intriguingly, common variants of *QTRT1* from genome‐wide analysis study (GWAS) in the GLGC were evidenced to influence serum TC and LDL‐C, but in our exome analysis rare mutations of *QTRT1* showed significant association with higher levels of TG, implying the complex role of *QTRT1* in the lipid metabolism and atherosclerosis, which deserves further research. *PPARD* has been clearly clarified to be involved in cholesterol metabolism [29] by encoding the lipid‐activated nuclear receptor and activate gene transcription, and deletion of the gene in ApoE knockout mice showed attenuated atherosclerosis [30]. In comparison, *CABP1* which participates in modulation of calcium channels [31] is rarely investigated the role in modulating LDL‐C. Similarly, no direct evidence was found to explain the role of *HLA‐E* in dyslipidemia, which encodes nonclassical histocompatibility leukocyte antigens Ib (HLA‐Ib) molecules involved in self‐surveillance and adaptive immune reaction [32]. Yet reduced expression of HLA‐DR, another form of HLA molecules was associated with increased triglycerides, which implicates that HLA‐associated dyslipidemia may result from alterations of the process of antigen presentation in monocytes [33]. The role of *TOE1* and *HPSE2* in dyslipidemia were also not mentioned in previous studies, with the limited knowledge that *TOE1* encodes an unconventional deadenylase and maintains telomerase activity [34], and *HPSE2* is determined as the culprit gene for an autosomal recessive disease named urofacial syndrome [35]. Of note, *CABP1* (OR = 22.19, *p* = 0.02), *HLA‐E* (OR = 22.19, *p* = 0.02), *TOE1* (OR = 33.6, *p* = 0.002) and *HPSE2* (OR = 11.1 in the PolyPhen set, *p* = 0.04) were of high effect size and recognized as novel genes potentially involved in residual dyslipidemic risk of EOCAD (Figure 1).

With regard to pathway of thrombosis, tendency of thrombosis and genes associated with shortened PT and APTT were neither seen in patients with EOCAD, which indicated that the role of coagulation and thrombosis in aggravating residual risk might be trivial. Comparatively, increased CRP, fibrinogen and total WBC count as well as neutrophil percentage were found in patients with EOCAD and *FBLN5, PCSK5, FAM161A, KLHL8, RALY, TOP2A, CHST14, C2orf73* were identified to increase these measurements of inflammation. These genes were not mentioned to associate with CAD in the CARDIoGRAMplusC4D but were mapped with cardiovascular and metabolic phenotypes in MGI, with *KLHL8*, *PCSK5*, and *C2orf73* mapped in the GLGC (Table S15). It is intriguing that several of these genes of interaction with inflammation are associated with the balance of extracellular matrix (ECM) and have been reported to be involved in various situations of inflammatory responses, such as *FBLN5* [36, 37], *PCSK5* [38], *CHST14* [39], and knockdown of TOP2A could alter the expression of matrix metalloproteinases (MMPs) [40] that are responsible for degradation of ECM and aggravating inflammation. However, the role of *CHST14* and *TOP2A* in initiating or enlarging immune responses of CAD seems to be possible, as CHST14 is responsible for synthesis of dermatan sulfate [39] and TOP2A mainly functions in DNA sequence rearrangements and cleavage [41]. Few evidence was found to support the roles of *FAM161A, KLHL8, RALY*, and *C2orf73* in inflammation. In summary, considering the effect size of *CHST14* (OR = 22.19, *p* = 0.02) and *KLHL8* (OR = 22.19, *p* = 0.02), these two novel genes might contribute to the residual inflammatory risks of EOCAD (Figure 1).

In our study, we found a series of genes that explain risk for non‐CRF‐EOCAD patients. We performed whole‐exome sequencing, which is the tool of rare variant association studies (RVASs) with the goal of discovering new pathogenic genes and further exploring the pathogenesis of EOCAD, differing from common variant association studies (CVASs) which allow for calculation of polygenic risk scores (PRS). In a CVAS, the frequencies of an individual common variant, usually noncoding, differ between cases and controls, establishing an association with a disease phenotype. Whereas, this study design does not typically identify the causal variant(s) or gene(s) [42]. In a RVAS, the aggregate frequencies of collections of rare variants in a gene differ between cases and controls, establishing the gene as being causal for a disease phenotype [42]. Discovery of novel causal genes by exome‐sequencing might provide additional value of a PRS which is based on genome‐sequencing, to evaluate disease susceptibility especially for certain race or ethnicity. It is of interest to compare and even combine these genes discovered with a PRS for net reclassification or predictive ability of EOCAD development and progression in future research.

## 5 Study Limitations

First, our sample size of the study population was limited to restricted number of patients with EOCAD but no conventional risk factors for CAD, which was due to restrained enrollment of total patients with EOCAD in given time period. Second, we included diabetes patients in the non‐CRF‐EOCAD group, which was due to the remarkably small sample size after excluding both diabetes and other conventional risk factors, which is too small to make the subsequent analysis feasibly carried out, and this issue would be addressed in future study enrolling larger study population. Besides, we found a minority of people in the control group suffering from myocardial infarction, who had undergone coronary angiography and had < 30% coronary stenosis. They might be patients who have been diagnosed with a special subtype of myocardial infarction named “MINOCA” (myocardial infarction with no obstructive coronary atherosclerosis). Future studies excluding possible MINOCA in control group would help drawing more precise conclusions. Third, the analytic section of the study was primarily conducted by gene‐based association analysis which is based on predictive genetic data sets and the mapping with other public database could provide certain support for the findings, which require further validation in population and experimental animals. Fourth, based on limited clinical data, we merely performed the phenotypic analysis related to inflammation with data of total counts of WBC, hsCRP and fibrinogen, which were not sufficient to fully represent the status of systemic and vascular inflammation in patients. Fifth, some of the genes which have been already mentioned in previous studies to be associated with dyslipidemia and inflammation might exert higher effect size, warranting further focus and elucidation.

## Author Contributions

Kang Yao contributed to conceptualization, methodology, writing review and editing. Junbo Ge contributed to conceptualization, methodology, and supervision. Runda Wu contributed to the conceptualization, data collection, investigation, and writing of the original draft. Ya Su contributed to the data collection, investigation, and writing of the original draft. Yuxiang Dai contributed to the methodology, investigation, and data curation. Juan Shen contributed to the data curation, software, and investigation. Wei Gao contributed to the investigation and data curation. Zheng Dong contributed to the investigation. Jianquan Liao contributed to the data collection and formal analysis. Yuanji Ma contributed to the data collection and curation.

Kang Yao and Yuxiang Dai are corresponding authors and are responsible for the overall content as guarantors.

## Conflicts of Interest

The authors declare no conflicts of interest.

## Supporting information

Supporting information.

## Data Availability

The data that support the findings of this study are available from the corresponding author upon reasonable request.
